# Pollution and Distribution of Microplastics in Grassland Soils of Qinghai–Tibet Plateau, China

**DOI:** 10.3390/toxics11010086

**Published:** 2023-01-16

**Authors:** Sumei Li, Ziyi Li, Jun Xue, Sha Chen, Hanbing Li, Jian Ji, Yixuan Liang, Jiaying Fei, Weiyi Jiang

**Affiliations:** 1 Department of Environment, Faculty of Environment and Life, Beijing University of Technology, Beijing 100124, China; 2 Key Laboratory of Beijing on Regional Air Pollution Control, Faculty of Environment and Life, Beijing University of Technology, Beijing 100124, China; 3 Solid Waste and Chemicals Management Center, Ministry of Ecology and Environment, Beijing 100029, China; 4 Graduate School of International Study, Yonsei University, Seoul 03722, Republic of Korea

**Keywords:** microplastics, grassland soil, pollution, Qinghai–Tibet Plateau, distribution

## Abstract

Microplastics (MPs) are plastic fragments with particle sizes smaller than 5 mm that have potentially harmful effects on ecosystems and human health. The soil environment is not only the source but also the sink of MPs. Thus, it is necessary to fully understand the pollution and distribution of MPs in soils. In this study, Qinghai Province, northeast of the Qinghai–Tibet Plateau, was selected as the research area, and 22 soil samples were collected and analyzed to study the levels and distribution characteristics of MPs in grassland soils. MPs were obtained from the soils by using density separation, and a laser confocal micro Raman spectrometer was used for MP identification. The results showed that MPs were detected in all of the soil samples. The total abundances of MPs ranged from 1125 to 1329 items/kg, with a mean abundance of 1202 items/kg. Various types, shapes, sizes, and colors of MPs were observed. Polyethylene terephthalate (PET) was the dominant polymer in all the grassland soil samples. The size range of 10–50 μm accounted for 50% of all identified MPs. Pellets were the dominant MP shape, and colored MPs accounted for 64% of all MPs. The results revealed the presence of large quantities of MPs in the grassland soils of remote areas as well. This study can act as a reference for further studies of MPs in terrestrial systems. At the end of the paper, the prospects and suggestions for pollution control by soil MPs are given.

## 1. Introduction

Plastics are widely and globally used high-molecular polymers. Due to the advantages of low cost, lightweight, durability, insulation, and stability, the production of plastics has continued to increase in the past few decades. The global plastics production was nearly 370 million tons in 2020. China, the North American Free Trade Agreement, the rest of Asia, and Europe accounted for 32%, 19%, 17%, and 15% of the total production, respectively [1]. The increase in plastic production also leads to large amounts of plastic waste being released into the environment. The annual production of mismanaged plastic waste is expected to reach 155–265 million tons by the year 2060 [2]. The plastic wastes exposed to the environment might be degraded into fine plastic particles or fragments under the influence of wind and sunlight. Among these particles, those with a particle size (or one-dimensional length) < 5 mm are defined as microplastics (MPs) [3,4]. The small particle size and large specific surface area of MPs facilitate their adsorption of harmful chemicals, such as persistent organic pollutants and heavy metals, thereby contributing to synergistic pollution that can affect the growth and regeneration of plants [5,6] and have potential harm to the global food chain and human health [7,8].

Current studies on MPs mainly focus on aqueous environments, such as oceans [9,10], lakes [11,12], and freshwater [13,14]. However, research on the abundances and fates of MPs in soils still remains limited, despite soils being considered a considerable plastic sink [15]. Many studies have demonstrated the potential for the accumulation of MPs to have adverse effects on soil physicochemical properties as well as animals, plants, and microorganisms in the soils [16,17,18,19]. Therefore, it is clear that further studies on MP pollution in soils are of great significance for fully understanding the impact of MPs on ecosystems.

Since Rillig [20] first evaluated the spatial distribution of MPs in the terrestrial ecosystem, there have been increasing numbers of related studies [21,22,23]. Recently, MPs were also detected in the soils of some remote areas, such as the Qinghai–Tibet Plateau [24]. However, related studies were mainly focused on agricultural soils, while only a few studies on grassland soils were located at relatively smaller horizontal scales [25,26,27]. There are vast grasslands on the Qinghai–Tibet Plateau, and animal husbandry is much more developed than agriculture. Therefore, studies on the pollution levels and distribution characteristics of MPs in the grassland soils in this area are of great reference value for the protection of the Qinghai–Tibet Plateau ecosystem.

In this study, we investigated the pollution and distribution of MPs in the grassland soils in Qinghai Province, northeast of the Qinghai–Tibet Plateau. Twenty-two sampling sites across the majority of Qinghai Province were selected. The aims of this study are to: (1) investigate the abundance and distribution of MPs in grasslands; (2) clarify the MPs’ distribution characteristics, including types, sizes, shapes, and colors; and (3) analyze the possible sources of MPs. The results of this study will improve our understanding of MPs in soils in remote areas and provide reference information for future MP pollution monitoring and risk assessment.

## 2. Materials and Methods

### 2.1. Study Areas and Samples Collection

**Figure 1:**
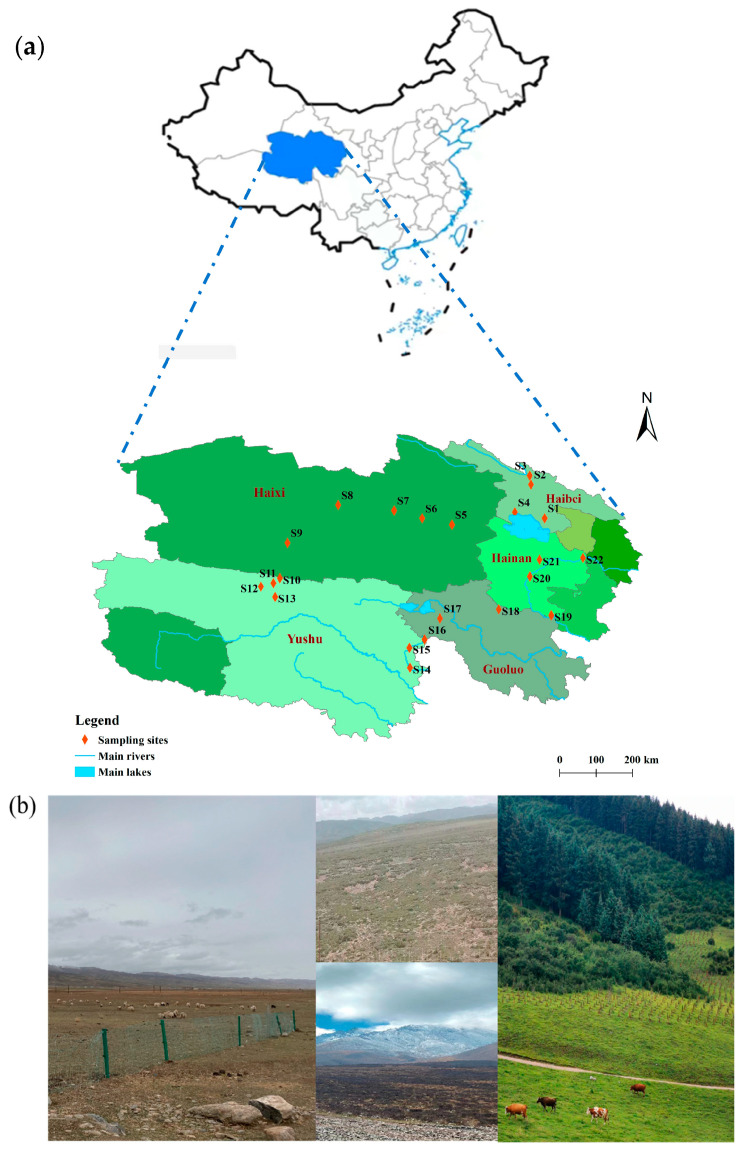
(**a**) Location of grassland soil sampling sites in Qinghai Province. (**b**) Photographs of grasslands in Qinghai Province.

As shown in Figure 1a, the study area (33°25′40″–38°10′54″ N, 93°35′58″–101°35′11″ E) is located in Qinghai Province, China. Qinghai Province is located in the northeast of the Qinghai–Tibet Plateau, with an average elevation of over 3000 m. This area is dominated by the plateau continental climate, which has characteristics of sufficient sunshine, strong radiation, and a large diurnal temperature difference. The average annual total radiation in Qinghai Province is 5860–7400 MJ/m^2^, the sunshine hours are 2336–3341 h, and the annual average temperature is −5.1–9.0 °C. The land area of Qinghai Province is 69.66 million hectares, 42.1334 million hectares of which is the natural grassland area, accounting for 60.48% of the total land area. According to the national unified classification, the grasslands in Qinghai Province can be divided into 9 grassland classes, 7 grassland subclasses, 28 grassland groups, and 173 grassland types. Among all kinds of grasslands, alpine meadows are the main type of natural grassland in Qinghai. The height of grass in Qinghai is low, the pastures are highly grazing-tolerant, and more than half of the grassland in Qinghai is dominated by Cyperaceae that are suitable for grazing. Therefore, the animal husbandry in Qinghai has been continuously developing in recent years. Some of the grassland photographs taken in Qinghai are shown in Figure 1b.

From July to August of 2021, twenty-two sampling sites were selected from the natural grasslands in Haibei Tibetan Autonomous Prefecture (HB, 4 sites), Haixi Mongolian Tibetan Autonomous Prefecture (HX, 5 sites), Yushu Tibetan Autonomous Prefecture (YS, 7 sites), Guoluo Tibetan Autonomous Prefecture (GL, 2 sites), and Hainan Tibetan Autonomous Prefecture (HN, 4 sites) in Qinghai Province. The specific geographic information of the sampling sites is shown in Table S1. At each sampling site, three sub-samples of topsoil (0–10 cm) were collected using a stainless-steel shovel and pooled as one. After removing large areas of visible waste (more than 10 cm), all the samples were packed in aluminum foil bags before being transported to the laboratory and stored in a refrigerator until analysis.

### 2.2. Microplastics Extraction

MPs in the grassland soil samples were separated by a density separation method, and the specific method was referred by Wan et al. [28], with some modifications. The soils were dried at 25 °C to a constant weight and filtered through a 5 mm stainless steel sieve. 20 g of the filtered soil sample was put into a clean beaker, and a saturated ZnCl_2_ solution (1.5 g/cm^3^) was added. The mixture was vigorously stirred for 5 min to ensure the soil and water were thoroughly mixed, and then the mixture was settled for 12 h. The supernatant was then transferred to another dry beaker. The procedure was repeated three times. Then 60 mL of H_2_O_2_ (30%) was added for digestion. The mixture was placed in a thermostatic shaker and shaken for 30 min at 65 °C at 100 rmp, then left for 24 h to allow the H_2_O_2_ to fully react with the organic matter. The obtained mixture was filtered through a 0.45 μm glass fiber membrane. The filter membrane was then placed in a clean glass Petri dish and dried at 50 °C for 24 h before observation.

### 2.3. Microplastic Identification and Data Analysis

MPs on the filter membrane were checked using a stereomicroscope (Chongqing COIC Industrial Co., Ltd. UB100-CV320, Chongqing, China) at a magnification of ×50. The images of all particles were obtained by a camera link to UopView software (UB100-CV320), and the particle size was determined as the longest dimension of images by the software. The shapes, sizes, colors, and abundance of the particles were recorded. The polymer types of particles were confirmed by a Laser Confocal Micro Raman Spectrometer (HORIBA, Kyoto, Japan) with a wavelength of 532 nm and a spectral scanning range of 50–3200 cm^−1^. The material spectra were compared with the reference polymer spectra provided by Biorad (Bio-Rad, Berkeley, CA, USA). The types of polymers were determined according to the degree of match between the obtained spectra and the characteristic peaks of specific polymers. A degree of matching over 90% was required to confirm a polymer type.

### 2.4. Data Analysis

A one-way analysis of variance (ANOVA) was used to determine the differences in the abundance of the microplastics in different regions, and *p* < 0.05 was considered to be statistically significant. A Spearman analysis was performed to study the correlation between the abundance of MPs and physicochemical properties in soil. The software Microsoft Excel 2017 (Microsoft Inc., Seattle, WA, USA), SPSS (SPSS Inc., Chicago, IL, USA), Origin 2018 (Origin Lab Inc., Northampton, MA, USA), and ArcGIS 10.7 (ESRI Inc., Redlands, CA, USA) were used for the data analysis and geographic mapping in this study.

### 2.5. Quality Assurance and Quality Control

The study implemented a series of precautions throughout the experimental process to avoid potential background contamination in the laboratory. Laboratory personnel were required to wear cotton laboratory clothing and nitrile gloves during the experiment. All laboratory instruments and supplies (measuring cylinders, glass beakers, glass Petri dishes, etc.) were carefully washed at least three times with Milli-Q water before use. The equipment and samples were covered with glass lids before and after each treatment step to minimize exposure periods. Blank experiments were conducted concurrently to investigate the effects of laboratory air on the experiment. The results showed that the average content of MPs in the air blank samples was 0.36 ± 0.55 items/L, which meant that the contamination from the laboratory was minimal and could be negligible.

## 3. Results and Discussion

### 3.1. The Abundance of Microplastics in Soil

**Figure 2:**
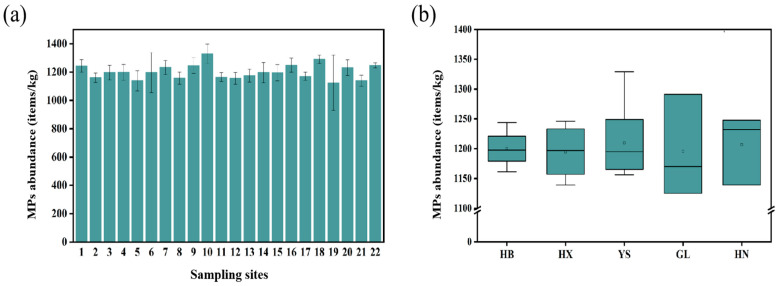
Distribution of the abundances of MPs in grassland soils of Qinghai Province. (**a**) MP abundances for the 22 sampling sites; (**b**) average MP abundance in the five regions.

As shown in Figure 2a, MPs were detected in all of the soil samples, indicating the prevalence of MP contamination in grassland soils in Qinghai Province. MPs were observed in all samples, ranging from 1125 to 1329 items/kg, with a mean abundance of 1202 items/kg. Table S1 provides the specific values of MP abundance at each sampling site. Overall, the different sampling sites showed similar abundances of MPs. This is probably because the soil samples were collected from similar types of land use, and thus the MPs’ pollution sources were similar.

Although there were differences in the abundance of MPs in soil samples among the five administrative regions, the differences were not very significant (*p* > 0.05). Figure 2b shows that the average abundances of MP in the HB, HX, YS, GL, and HN regions were 1200, 1194, 1209, 1231, and 1186 items/kg, respectively. These differences in MPs abundance among the different regions could be attributed to the influences of certain factors, such as population density [29]. However, it was found that there was no significant correlation between the abundance of MPs and the population density (*p* > 0.05) in this study. It may be due to the fact that most of the sampling sites were far from densely populated areas. Another reason might be the unevenness of the sampling sites in a large sampling area with sparse but highly diverse population densities [25].

Except for the abundance of MPs in soil samples, the pH and TOC of soils were also determined, and the relationship among them was discussed using Spearman correlation. The results revealed that there was no significant correlation between the abundance of MPs and the pH and TOC of the soils (*p* = 0.118 and *p* = 0.400) in this study.

Table 1 summarizes MP contamination in different soil types in the different regions. In general, there were relatively lower abundances of MPs in Qinghai Province compared with those found by other studies in other areas. The maximum MPs abundance of 1329 items/kg identified in the present study was far lower than that found in Wuhan (6.9 × 10^5^ items/kg), Beijing (13,752 items/kg), and Shaanxi (3410 items/kg) [30,31,32]. This may be because Qinghai Province is a relatively remote region and the sampling sites are located in grasslands, which experience relatively low intensities of human activities. In addition, MPs have also been found in other remote areas of China, such as Yunnan [33] and Xinjiang [34] (Table 1). These results indicate that soil MP contamination has become ubiquitous across China. The presence of MPs may pose a threat to the soil ecosystem. From one perspective, it is because of the harmful substances contained in the microplastics, such as bisphenol A, phthalates, polychlorinated biphenyl ethers, and heavy metals [35,36]. These substances in plastics may be released into the environment under the influence of UV radiation, temperature, soil acidity and alkalinity, and soluble soil organic matter, then enter the soil through leaching, posing an adverse impact on the soil ecosystem, and even enter the food chain through plant uptake, thus threatening human health [36,37,38]. Alternatively, MPs could adsorb other pollutants in the soils, such as heavy metals and organic pollutants, thus forming compound pollution, which may pose a greater threat to the ecological environment than the single MPs [39,40]. Therefore, relevant administrations should formulate corresponding policies for the management of MPs. Various measures should be taken during the use of plastic products, resource recovery, and utilization of plastic wastes. Meanwhile, degradable plastics should be promoted to reduce the amount of plastic waste released into the environment.

### 3.2. The Components and Possible Sources of Microplastics in Soils

**Figure 3:**
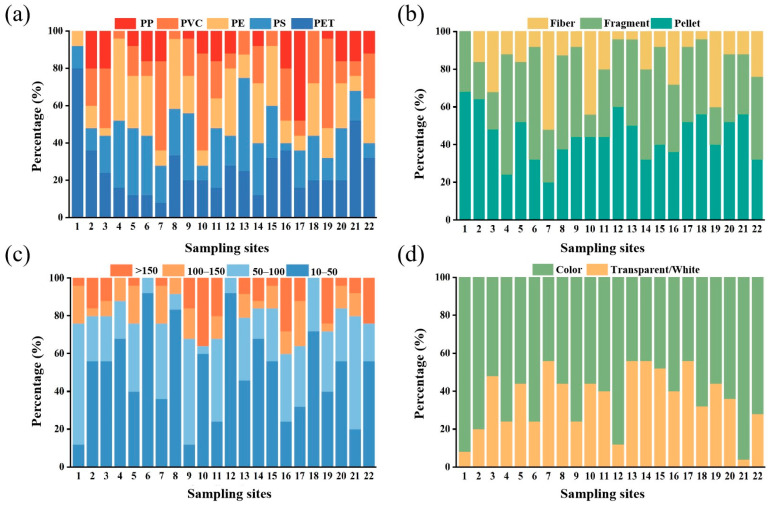
MPs in the grassland soils of Qinghai Province. (**a**) type, (**b**) shape, (**c**) size, (**d**) color.

**Figure 4:**
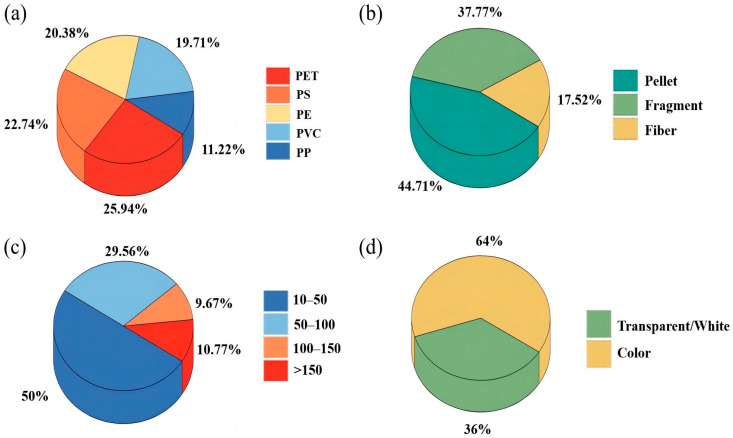
Polymer type (**a**), shape (**b**), size (**c**), and color (**d**) distribution of MPs in the soils of the sampling sites in Qinghai.

We identified five types of MP polymers in the grassland soils by Raman spectroscopy, including PE, PET, PP, PVC, and PS. The Raman spectra of the five MPs are shown in Figure S1. These polymers are also the most common types of MPs in the environment because they possess a carbon backbone that is resistant to hydrolytic and enzymatic degradation. As such, microorganisms are generally unable to assimilate and mineralize these polymers, leading to the accumulation of these materials in the environment [15]. As shown in Figure 3a, the contribution of each polymer type to MP contamination varied among the different sampling sites. Overall, PET was the dominant polymer type, with a contribution of 25.9%, followed by PS, PE, PVC, and PP, accounting for 22.7%, 20.4%, 19.7%, and 11.2%, respectively (Figure 4a). Previous studies found that the main polymer types of MPs varied according to different soil uses. For example, PE has been identified as the dominant MP polymer in most studies on agricultural soils [6,31], which could possibly be attributed to the widespread use of PE as plastic mulch in farmland.

There was wide variation in MP types in soils among the different regions, which could be closely related to the different sources of plastic. In general, sources of MPs in soils mainly include landfill disposal of plastic wastes, soil amendments, the application of sewage sludge to land, the application of compost and organic fertilizer, residues of agricultural mulching films, tire wear and tear, and atmospheric deposition [31,41]. Plastic mulch and sewage sludge composting were the main sources of MP pollution in farmlands [39,42]. Plastic dust-proof nets might be a major contributor to MP contamination in construction areas [31]. For grassland soils, animal husbandry, tourism, and atmospheric transport could be the main sources of MPs. MPs have been found in sheep feces by Beriot et al. [43], suggesting that grazing activities might contribute to MP contamination of grasslands. In addition, the large and uneven surface of plant leaves could highly capture atmospheric particles (including MPs) and become an important source of absorption of various atmospheric pollutants [44,45]. The MPs trapped on the plant leaves can enter the soil through fallen leaves or precipitation [44]. Therefore, atmospheric transport is also an important source of grassland MPs.

### 3.3. Shape, Size, and Color Characteristics of MP in the Grassland Soils

**Figure 5:**
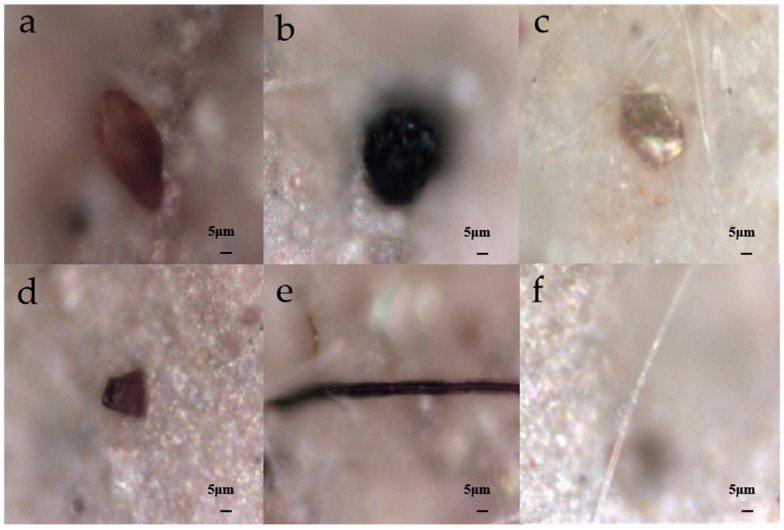
Photographs of typical MPs in the shapes of pellets (**a**,**b**), fragments (**c**,**d**), fibers (**e**,**f**).

The MPs in the grassland soils of Qinghai Province showed three major shapes: pellet, fragment and fiber. Figure 5 shows the optical microscope images of typical MPs types. Among the three shapes, the percentage of pellets was the highest (44.71%), followed by fragments (37.77%), and fibers (17.52%) (Figure 4b). These three shapes of MPs were very common in the soil environment, but their specific gravities varied to a certain extent in different studies (Table 1). Pellets had the highest concentration at site 1, reaching 68%. It should be noted that since pellets are more mobile than other shapes, they more readily migrate downward or with rainwater runoff [46]. Among all of the sampling sites, site 7 had the highest proportion of fibers (52%). Compared with pellets, fibers are more likely to be entangled with the soil matrix and have different interactions with soil biological communities [47]. For fragments, the highest proportion of about 64% appeared in site 4, which was close to Qinghai Lake, and the plastic waste brought by tourism might be the main source of MPs.

The size distribution of MPs is presented in Figure 3c and four categories were classified according to the particle sizes (10–50 μm, 50–100 μm, 100–150 μm, and >150 μm). The results showed that the abundance of MPs increased with the decrease in sizes in general (Figure 4c), which was consistent with the observation of previous studies [21,23]. The sizes of the MPs that were found in this study were mostly below 150 μm (89.23%). The abundances of the small-sized MPs were higher than those of the large-sized MPs, indicating that the small-sized MPs might be decomposed from the large-sized MPs [26]. The sizes of MPs in the grassland soil samples of this study were smaller than those in previous studies [24,25], which might be related to the climate of Qinghai Province, which endures long sunshine hours, strong radiation, and is dry and windy. Therefore, MPs in grassland soils are also experiencing strong weathering and UV radiation, making them more prone to being broken up from large sizes into small ones. In addition, a portion of the MPs might come from animal feces. After digestion by gastric juice, large-sized MPs might also become smaller ones. Smaller MPs might be more readily absorbed by organisms. Li et al. [48,49] exposed lettuce and wheat to PS of different particle sizes (0.2, 1.0, 2.0, 5.0, and 7.0 μm), and the experimental results showed that 0.2 μm PS was more likely to enter the root cell interstices. A study of MPs in Chinese marine shellfish found that MPs with particle size less than 250 μm accounted for 84% of the total [50]. Therefore, MPs of small sizes may pose a more serious threat to the ecological environment.

It was found that the colors of MPs in the grassland soils in this study included white, wine red, transparent, green, black, gray, and brown. In order to facilitate statistics, the MPs in this study were simply divided into transparent/white and colored MPs (Figure 3d), which accounted for 36% and 64%, respectively (Figure 4d). Normally, colored plastics have greater attractiveness and longevity, especially those made for tourism [51,52]. Therefore, the colored plastics in this study might come from daily colored plastic necessities, such as colored bags, packages, and bottles. Colored MPs are more attractive to birds and other animals, which take them as food due to their bright colors [53]. Alternatively, pigments (e.g., titanium dioxide and iron oxides) used as plastic additives might be gradually released from plastics and subsequently transferred to other environmental media and even organisms [15,36,54]. Hence, colored MPs in the grassland soils might pose a greater environmental threat and cause higher ecological risks in the Qinghai–Tibet Plateau.

### 3.4. Impacts of Microplastics on Soil

Due to the low light and relatively anaerobic environment in the soil, MPs might exist in soils for decades or more. Under long-term coexistence conditions, MPs could change the physical and chemical properties of the soil. It has been reported that the effect of MPs on the soil properties depends mainly on the types of MPs. For example, PET could significantly reduce the density of soil particles, resulting in a significant increase in the soil water storage capacity and evapotranspiration, while PE did not trigger as significant a decrease in the bulk density of the soil as PET [16,17]. In addition, MPs might also affect the chemical properties of soils. Dong et al. [55] found that a higher content of plastic film residues would lead to a decrease in pH, organic matter, alkaline hydrolyzed nitrogen, available phosphorus, and available potassium in soil. These deviations from a natural state indicate that the presence of microplastics could pose a potential threat to soil ecosystems.

Moreover, MPs also have potential negative effects on soil microorganisms. The pollution of soil MPs could also disturb the soil microbial community and affect enzyme activity. Liu et al. [56] found that PP (28%, *w*/*w*) had a significant stimulatory effect on fluorescein diacetate hydrolase activity, leading to enhanced activity of soil microorganisms in sandy loam soils. However, de Souza Machado et al. [16] found that polyacrylic and polyester fibers (0.1%, *w*/*w*) reduced the activity of microorganisms in sandy loam. Wang et al. [57] reported that plastic film residues reduced enzyme activity and microbial community diversity in soils, which might be caused by the release of phthalic acid esters (PAEs) from plastic film residues. As far as we know, many other additives, such as flame retardants, antioxidants, lubricants, coloring agents, etc., are used to fabricate many kinds of plastics. Therefore, the plastic additives that lixiviate due to erosion should be ignored, and further research should be focused on the combined contamination of MPs and their additives in the soil environment.

## 4. Conclusions and Prospects

We collected 22 grassland soil samples from Qinghai Province to study the abundance and distribution characteristics of MPs. The results showed that MPs were detected in all soil samples collected in the study area. The abundances of MPs ranged from 1125 to 1329 items/kg, with a mean abundance of 1202 items/kg. PET, PS, and PE were the dominant polymer types for the identified MPs. Small size, pellets, fragments, and color were the main contamination characteristics of MPs observed. This study showed that the grassland soils in Qinghai Province were universally contaminated by MPs. The results of this study provide an important reference for future studies on MP pollution in terrestrial ecosystems of the Qinghai–Tibet Plateau.

The current research showed that the MP pollution in the grassland soils of Qinghai Province could not be ignored. Therefore, measures should be taken to mitigate the MPs’ pollution. At present, the Chinese government has formulated many regulations to restrict the production, sale, use, and recycling of some plastic products. However, it is necessary to strengthen supervision to ensure the implementation of the related policies and regulations. Furthermore, the related enterprises should take sustainable use of plastics as part of their social responsibility. Sustainable and environmentally friendly materials should be developed to replace nondegradable plastics. Packaging materials and technology should be improved to reduce the release of plastics to the environment. At last, public awareness and responsibility to reduce the use and promote the recycling of plastics should be raised.

## Figures and Tables

**Table 1:** Detailed information on soil microplastics in previous studies from other regions in comparison to this study.

Country	Location	Soil Types	Abundances (Items/kg)	Polymer Types	Main Size and Shape	Reference
China	Tibetan Plateau	Vacant land	5–340	polyvinyl chloride (PVC), polyethylene (PE), polypropylene (PP), polyethylene terephthalate (PET), polyvinyl alcohol (PVAL)	Size: <500 μm (66.2%); Shape: fiber (45%), fragment (32%),	[24]
China	Qinghai–Tibet Plateau	Farmland and Grassland	0–260	PE, polyamide (PA), polystyrene (PS), PP	Size: <500 μm (shallow soil: 66%; deep soil: 79%) Shape: film (shallow soil: 36%; deep soil: 41%)	[25]
China	Qinghai	Agricultural	240–3660	N.A.	Size: <0.5 mm (50%), 0.5–1 mm (33%) Shape: film (67%),	[26]
China	Wuhan	Suburban	2.2 × 10^4^–6.9 × 10^5^	PE, PP, PS, and PA	Size: 10–50 μm (46.1%), 50–100 μm (35.3%) Shape: fragment (52%)	[30]
China	Beijing	Site	272–13,752	PE and PP	Size: >1000 μm (60.43%); Shape: fiber (42.88%), particles (42.05%)	[31]
China	Shaanxi	Agricultural	1430–3410	PS, PE, PP, PVC, PET, high-density polyethylene (HDPE)	Size: 0–0.49 mm (81%) Shape: fiber (49%)	[32]
China	Yunnan	Farmland	0.9 × 103–40.8 × 10^3^	N.A.	Size: <500 μm (89.3%) Shape: fragment (78.3%),	[33]
China	Xinjiang	Farmland	80–1071	PE	N.A.	[34]
China	Qinghai	Grassland	1125–1329	PET, PS, PE, PVC, and PP	Size: 10–50 μm (50%) Shape: pellet (44.17%)	This study

## Data Availability

Not applicable.

## Supplementary Materials

The following supporting information can be downloaded at: https://www.mdpi.com/article/10.3390/toxics11010086/s1. Table S1: Specific information of each sampling site in Qinghai Province; Figure S1: Raman spectra of each polymer (a: PET, b: PS, c: PE, d: PVC, e: PP).

## References

[1] (2020). PlasticEurope.cPlastics–the Facts 2021: An Analysis of European Plastics Production, Demand and Waste Data. https://plasticseurope.org/knowledge-hub/plastics-the-facts-2021/.

[2] Lebreton L., Andrady A. (2019). Future scenarios of global plastic waste generation and disposal. Palgrave. Commun..

[3] Andrady A.L. (2011). Microplastics in the marine environment. Mar. Pollut. Bull..

[4] Thompson R.C., Olsen Y., Mitchell R.P., Davis A., Rowland S.J., John A.W.G., Mcgonigle D., Russell A.E. (2004). Lost at sea: Where is all the plastic?. Science.

[5] Lang M., Yu X., Liu J., Xia T., Wang T., Jia H., Guo X. (2020). Fenton aging significantly affects the heavy metal adsorption capacity of polystyrene microplastics. Sci. Total Environ..

[6] Zhou B., Wang J., Zhang H., Shi H., Fei Y., Huang S., Tong Y., Wen D., Luo Y., Barceló D. (2020). Microplastics in agricultural soils on the coastal plain of Hangzhou Bay, east China: Multiple sources other than plastic mulching film. J. Hazard. Mater..

[7] Leslie H.A., van Velzen M.J.M., Brandsma S.H., Vethaak A.D., Garcia-Vallejo J.J., Lamoree M.H. (2022). Discovery and quantification of plastic particle pollution in human blood. Environ. Int..

[8] Ragusa A., Svelato A., Santacroce C., Catalano P., Notarstefano V., Carnevali O., Papa F., Rongioletti M.C.A., Baiocco F., Draghi S. (2021). Plasticenta: First evidence of microplastics in human placenta. Environ. Int..

[9] Warrier A.K., Kulkarni B., Amrutha K., Jayaram D., Valsan G., Agarwal P. (2022). Seasonal variations in the abundance and distribution of microplastic particles in the surface waters of a Southern Indian Lake. Chemosphere.

[10] Courtene-Jones W., Quinn B., Gary S.F., Mogg A.O.M., Narayanaswamy B.E. (2017). Microplastic pollution identified in deep-sea water and ingested by benthic invertebrates in the Rockall Trough, North Atlantic Ocean. Environ. Pollut..

[11] Zhang Q., Liu T., Liu L., Fan Y., Rao W., Zheng J., Qian X. (2021). Distribution and sedimentation of microplastics in Taihu Lake. Sci. Total Environ..

[12] Bashir A., Hashmi I. (2022). Detection in influx sources and estimation of microplastics abundance in surface waters of Rawal Lake, Pakistan. Heliyon.

[13] Fu Z., Wang J. (2019). Current practices and future perspectives of microplastic pollution in freshwater ecosystems in China. Sci. Total Environ..

[14] Ding R., Tong L., Zhang W. (2021). Microplastics in Freshwater Environments: Sources, Fates and Toxicity. Water. Air. Soil Pollut..

[15] Ng E.L., Huerta Lwanga E., Eldridge S.M., Johnston P., Hu H.W., Geissen V., Chen D. (2018). An overview of microplastic and nanoplastic pollution in agroecosystems. Sci. Total Environ..

[16] de Souza Machado A.A., Lau C.W., Till J., Kloas W., Lehmann A., Becker R., Rillig M.C. (2018). Impacts of microplastics on the soil biophysical environment. Environ. Sci. Technol..

[17] de Souza Machado A.A., Lau C.W., Kloas W., Bergmann J., Bachelier J.B., Faltin E., Becker R., Görlich A.S., Rillig M.C. (2019). Microplastics can change soil properties and affect plant performance. Environ. Sci. Technol..

[18] Ju H., Zhu D., Qiao M. (2019). Effects of polyethylene microplastics on the gut microbial community, reproduction and avoidance behaviors of the soil springtail, Folsomia candida. Environ. Pollut..

[19] Kim S.W., An Y.J. (2019). Soil microplastics inhibit the movement of springtail species. Environ. Int..

[20] Rillig M.C. (2012). Microplastic in terrestrial ecosystems and the soil?. Environ. Sci. Technol..

[21] Scheurer M., Bigalke M. (2018). Microplastics in Swiss floodplain soils. Environ. Sci. Technol..

[22] Garcés-Ordóñez O., Castillo-Olaya V.A., Granados-Briceño A.F., Blandón García L.M., Espinosa Díaz L.F. (2019). Marine litter and microplastic pollution on mangrove soils of the Ciénaga Grande de Santa Marta, Colombian Caribbean. Mar. Pollut. Bull..

[23] Liu M., Lu S., Song Y., Lei L., Hu J., Lv W., Zhou W., Cao C., Shi H., Yang X. (2018). Microplastic and mesoplastic pollution in farmland soils in suburbs of Shanghai, China. Environ. Pollut..

[24] Yang L., Kang S., Wang Z., Luo X., Guo J., Gao T., Chen P., Yang C., Zhang Y. (2022). Microplastic characteristic in the soil across the Tibetan Plateau. Sci. Total Environ..

[25] Feng S., Lu H., Liu Y. (2021). The occurrence of microplastics in farmland and grassland soils in the Qinghai-Tibet plateau: Different land use and mulching time in facility agriculture. Environ. Pollut..

[26] Lang M., Wang G., Yang Y., Zhu W., Zhang Y., Ouyang Z., Guo X. (2022). The occurrence and effect of altitude on microplastics distribution in agricultural soils of Qinghai Province, northwest China. Sci. Total Environ..

[27] Zhang H., Huang Y., An S., Li H., Deng X., Wang P., Fan M. (2022). Land-use patterns determine the distribution of soil microplastics in typical agricultural areas on the eastern Qinghai-Tibetan Plateau. J. Hazard. Mater..

[28] Wan Y., Wu C., Xue Q., Hui X. (2019). Effects of plastic contamination on water evaporation and desiccation cracking in soil. Sci. Total Environ..

[29] Zhou Y., He G., Jiang X., Yao L., Ouyang L., Liu X., Liu W., Liu Y. (2021). Microplastic contamination is ubiquitous in riparian soils and strongly related to elevation, precipitation and population density. J. Hazard. Mater..

[30] Zhou Y., Liu X., Wang J. (2019). Characterization of microplastics and the association of heavy metals with microplastics in suburban soil of central China. Sci. Total Environ..

[31] Chen Y., Wu Y., Ma J., An Y., Liu Q., Yang S., Qu Y., Chen H., Zhao W., Tian Y. (2021). Microplastics pollution in the soil mulched by dust-proof nets: A case study in Beijing, China. Environ. Pollut..

[32] Ding L., Zhang S., Wang X., Yang X., Zhang C., Qi Y., Guo X. (2020). The occurrence and distribution characteristics of microplastics in the agricultural soils of Shaanxi Province, in north-western China. Sci. Total Environ..

[33] Huang B., Sun L., Liu M., Huang H., He H., Han F., Wang X., Xu Z., Li B., Pan X. (2020). Abundance and distribution characteristics of microplastic in plateau cultivated land of Yunnan Province, China. Environ. Sci. Pollut. Res..

[34] Huang Y., Liu Q., Jia W., Yan C., Wang J. (2020). Agricultural plastic mulching as a source of microplastics in the terrestrial environment. Environ. Pollut..

[35] Rochman C.M., Manzano C., Hentschel B.T., Simonich S.L.M., Hoh E. (2013). Polystyrene plastic: A source and sink for polycyclic aromatic hydrocarbons in the marine environment. Environ. Sci. Technol..

[36] Teuten E.L., Saquing J.M., Knappe D.R.U., Barlaz M.A., Jonsson S., Björn A., Rowland S.J., Thompson R.C., Galloway T.S., Yamashita R. (2009). Transport and release of chemicals from plastics to the environment and to wildlife. Phil. Trans. R. Soc. B..

[37] Xu S.Y., Zhang H., He P.J., Shao L.M. (2011). Leaching behaviour of bisphenol A from municipal solid waste under landfill environment. Environ. Technol..

[38] Sun J., Wu X., Gan J. (2015). Uptake and metabolism of phthalate esters by edible plants. Environ. Sci. Technol..

[39] Ramos L., Berenstein G., Hughes E.A., Zalts A., Montserrat J.M. (2015). Polyethylene film incorporation into the horticultural soil of small periurban production units in Argentina. Sci. Total Environ..

[40] Zhang S., Han B., Sun Y., Wang F. (2020). Microplastics influence the adsorption and desorption characteristics of Cd in an agricultural soil. J. Hazard. Mater..

[41] Kumar M., Xiong X., He M., Tsang D.C.W., Gupta J., Khan E., Harrad S., Hou D., Ok Y.S., Bolan N.S. (2020). Microplastics as pollutants in agricultural soils. Environ. Pollut..

[42] Briassoulis D., Babou E., Hiskakis M., Kyrikou I. (2015). Analysis of long-term degradation behaviour of polyethylene mulching films with pro-oxidants under real cultivation and soil burial conditions. Environ. Sci. Pollut. Res..

[43] Beriot N., Peek J., Zornoza R., Geissen V., Huerta Lwanga E. (2021). Low density-microplastics detected in sheep faeces and soil: A case study from the intensive vegetable farming in Southeast Spain. Sci. Total Environ..

[44] Bi M., He Q., Chen Y. (2020). What roles are terrestrial plants playing in global microplastic cycling?. Environ. Sci. Technol..

[45] Rindy J.E., Ponette-González A.G., Barrett T.E., Sheesley R.J., Weathers K.C. (2019). Urban trees are sinks for soot: Elemental carbon accumulation by two widespread oak species. Environ. Sci. Technol..

[46] O’Connor D., Pan S., Shen Z., Song Y., Jin Y., Wu W.M., Hou D. (2019). Microplastics undergo accelerated vertical migration in sand soil due to small size and wet-dry cycles. Environ. Pollut..

[47] Galloway T.S., Cole M., Lewis C. (2017). Interactions of microplastic debris throughout the marine ecosystem. Nat. Ecol. Evol..

[48] Li L., Luo Y., Peijnenburg W.J.G.M., Li R., Yang J., Zhou Q. (2020). Confocal measurement of microplastics uptake by plants. MethodsX.

[49] Li L., Zhou Q., Yin N., Tu C., Luo Y. (2019). Uptake and accumulation of microplastics in an edible plant (in Chinese). Chin. Sci. Bull..

[50] Li J., Yang D., Li L., Jabeen K., Shi H. (2015). Microplastics in commercial bivalves from China. Environ. Pollut..

[51] Rodrigues M.O., Abrantes N., Gonçalves F.J.M., Nogueira H., Marques J.C., Gonçalves A.M.M. (2018). Spatial and temporal distribution of microplastics in water and sediments of a freshwater system (Antuã River, Portugal). Sci. Total Environ..

[52] Zhang K., Shi H., Peng J., Wang Y., Xiong X., Wu C., Lam P.K.S. (2018). Microplastic pollution in China’s inland water systems: A review of findings, methods, characteristics, effects, and management. Sci. Total Environ..

[53] Costa M.F., Ivar Do Sul J.A., Silva-Cavalcanti J.S., Araújo M.C.B., Spengler Â., Tourinho P.S. (2010). On the importance of size of plastic fragments and pellets on the strandline: A snapshot of a Brazilian beach. Environ. Monit. Assess..

[54] Lithner D., Damberg J., Dave G., Larsson Å. (2009). Leachates from plastic consumer products-Screening for toxicity with Daphnia magna. Chemosphere.

[55] Dong H., Liu T., Han Z., Sun Q., Li R. (2015). Determining time limits of continuous film mulching and examining residual effects on cotton yield and soil properties. J. Environ. Biol..

[56] Liu H., Yang X., Liu G., Liang C., Xue S., Chen H., Ritsema C.J., Geissen V. (2017). Response of soil dissolved organic matter to microplastic addition in Chinese loess soil. Chemosphere.

[57] Wang J., Lv S., Zhang M., Chen G., Zhu T., Zhang S., Teng Y., Christie P., Luo Y. (2016). Effects of plastic film residues on occurrence of phthalates and microbial activity in soils. Chemosphere.

