# Supplementary material for: Pollution and Distribution of Microplastics in Grassland Soils of Qinghai–Tibet Plateau, China
Source: Toxics. 2023 Jan 16;11(1):86. doi: 10.3390/toxics11010086 (PMC9860952; doi:10.3390/toxics11010086)
Supplement: Supplementary file 1 [file toxics-11-00086-s001.zip › toxics-2105926-supplementary.pdf]

toxics

Pollution and Distribution of Microplastics in Grassland Soils of Qinghai–Tibet Plateau, China

Sumei Li[1,2], Ziyi Li[1,2], Jun Xue[3], Sha Chen[1,2]*, Hanbing Li[1,2], Jian Ji[1,2], Yixuan Liang[1,2], Jiaying Fei[1,2], Jiang Weiyi[4]

Table S1. Specific information of each sampling site in Qinghai Province.

Sampling number	Cities	Soil types	Altitude（m）	Longitude	Latitude	Abundances（items/kg）	pH	TOC (%)
S1	Haibei Tibetan Autonomous Prefecture	Umbrisols	3363	100°38'12"	37°7'31"	1244	9.28	7.52
S2	Haibei Tibetan Autonomous Prefecture		3791.7	100°17'53"	37°57'48"	1161	8.25	5.207
S3	Haibei Tibetan Autonomous Prefecture		3096.8	100°15'57"	38°10'54"	1197	7.94	8.172
S4	Haibei Tibetan Autonomous Prefecture		3233.9	99°53'56"	37°16'21"	1198	8.88	7.428
S5	Haixi Mongolian Tibetan Autonomous Prefecture		2971.8	98°20'5"	36°57'47"	1139	8.6	8.006
S6	Haixi Mongolian Tibetan Autonomous Prefecture		2852.9	97°35'51"	37°7'19"	1197	9	1.082
S7	Haixi Mongolian Tibetan Autonomous Prefecture		2816.3	96°54'5"	37°18'58"	1233	8.53	7.055

S8	Haixi Mongolian Tibetan Autonomous Prefecture		3182.1	95°30'43"	37°27'4"	1157	9.41	1.935
S9	Haixi Mongolian Tibetan Autonomous Prefecture		2776.7	94°15'29"	36°30'44"	1246	9.29	5.36
S10	Yushu Tibetan Autonomous Prefecture		4797.6	94°4'10"	35°38'26"	1329	9.65	3.076
S11	Yushu Tibetan Autonomous Prefecture		4605.5	93°54'36"	35°31'7"	1165	9.76	7.505
S12	Yushu Tibetan Autonomous Prefecture		4462.3	93°35'58"	35°26'1"	1156	8.58	4.12
S13	Yushu Tibetan Autonomous Prefecture		4425.7	93°57'11"	35°10'44"	1175	9.67	3.826
S14	Yushu Tibetan Autonomous Prefecture		4267.2	97°17'46"	33°25'40"	1197	9.58	3.458
S15	Yushu Tibetan Autonomous Prefecture		4495.8	97°17'2"	33°55'18"	1195	8.37	8.032
S16	Yushu Tibetan Autonomous Prefecture		4831.1	97°39'26"	34°7'38"	1249	9.64	3.237
S17	Guoluo Tibetan Autonomous Prefecture		4264.2	98°2'26"	34°38'59"	1170	8.52	3.025

S18	Guoluo Tibetan Autonomous Prefecture		4456.2	99°29'42"	34°52'18"	1291	8.77	3.016
S19	Guoluo Tibetan Autonomous Prefecture		3419.9	100°48'6"	34°43'36"	1125	9.02	1.985
S20	Hainan Tibetan Autonomous Prefecture		2650.3	100°15'58"	35°41'7"	1232	9.18	2.819
S21	Hainan Tibetan Autonomous Prefecture		2953.5	100°30'22"	36°5'55"	1139	9.25	2.677
S22	Hainan Tibetan Autonomous Prefecture		2237.2	101°35'11"	36°8'23"	1248	9.67	5.902

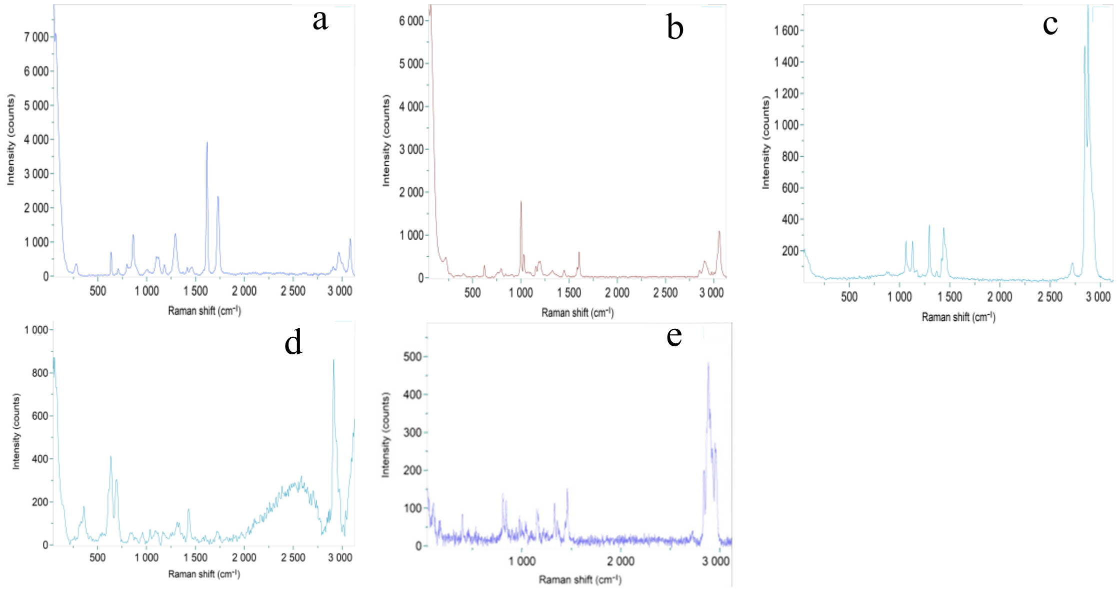

Figure S1. Raman spectra of each polymer. (a: PET, b: PS, c: PE, d:PVC, e: PP)
